# Expression of the cancer testis antigen IGF2BP3 in colorectal cancers; IGF2BP3 holds promise as a specific immunotherapy target

**DOI:** 10.18632/oncoscience.174

**Published:** 2015-07-01

**Authors:** HMC Shantha Kumara, Daniel Kirchoff, Otavia L. Caballero, Tao Su, Aqeel Ahmed, Sonali AC. Herath, Linda Njoh, Vesna Cekic, Andrew J. Simpson, Carlos Cordon-Cardo, Richard L. Whelan

**Affiliations:** ^1^ Division of Colon and Rectal Surgery, Department of Surgery, Mount Sinai Roosevelt Hospital Center, Suite 7B, New York, USA; ^2^ Ludwig Institute for Cancer Research Ltd, New York Branch of Human Cancer Immunology at Memorial Sloan-Kettering, New York, USA; ^3^ Herbert Irving Comprehensive Cancer Center, Columbia University, New York, USA; ^4^ Ludwig Institute for Cancer Research, New York, USA; ^5^ Orygen Biotecnologia S.A., São Paulo, Brazil; ^6^ Department of Pathology, Annenberg Building, New York, USA; ^7^ Icahn School of Medicine at Mount Sinai, New York, USA

**Keywords:** IGF2BP3, colorectal cancer, immunotherapy target

## Abstract

**Introduction:**

IGF2BP3 (IMP3) is a mRNA binding protein that regulates IGF2 translation and function during embryogenesis. Because IGF2BP3 is undetectable in adult human tissues except the testis, and increased IGF2BP3 expression has been noted in several cancers, it is considered a cancer testis (CT) protein. IGF2BP3 mRNA expression in colorectal cancers (CRC) has not been well studied. This study's aim was to quantitatively assess IGF2BP3 mRNA expression in CRC and, thus, determine if IGF2BP3 has potential as a vaccine target.

**Method:**

Data were collected prospectively from CRC patients in an IRB-approved tissue and data bank. Total RNA was isolated and purified from tumor and normal colonic tissue samples and cDNA synthesized. IGF2BP3 expression was analyzed by quantitative PCR (QPCR). Expression levels of IGF2BP3 in tumors and testis were determined and compared. Tumors with levels greater than 0.1% or more of the testis levels were considered positive. Analysis of IGF2BP3 protein expression by immunohistochemistry (IHC) in tumor and normal tissues was also performed.

**Results:**

A total of 84 paired tumor and normal tissue specimens were assessed from patients with Stage 2 and 3 CRC; 43% of tumors had IGF2BP3 mRNA expression levels greater than 0.1 % of that of testis and were considered positive. The median tumor expression level was higher in women (p=0.042). No correlation was found between IGF2BP3 mRNA expression and tumor stage or lymph node involvement. IHC was carried out on paired tumor and normal tissue sections from 46 patients; IGF2BP3 staining was noted in 50% of the tumor sections and in 5% of the normal tissue sections.

**Discussion:**

IGF2BP3 mRNA was over expressed in 43% of the tumors whereas the protein was noted in 50% of samples. No correlation between mRNA expression and disease severity was noted. This protein holds promise as a vaccine target, however, a larger study that assesses a more diverse population of patients (Stage 1-4) as well as a study of preoperative serum samples for auto-antibodies to IGF2BP3 are needed to pursue this concept.

## INTRODUCTION

Surgery remains the mainstay of treatment for patients with colorectal cancer. The last two decades have seen the development of new chemotherapeutic agents and adjuvant and neoadjuvant treatment regimens that have notably improved the long term outcome of these patients. Despite these advances, over 40% of patients who undergo “curative” surgery will progress and develop metastatic disease [1]. Therefore, the search for new anti-cancer agents and therapies is warranted.

For many years, numerous investigators around the world have been pursuing the concept of cancer vaccines. The idea of harnessing the patient's immune system to recognize and attack tumor cells is very attractive and likely to be associated with minimal toxicity when compared to conventional chemotherapy agents. Much progress has been made over the past 40 years in regards to our understanding of how the immune system functions and also how the tumor evades immune detection or inhibits the immune response. A very important advance was the approval for clinical use of antibodies that block immune checkpoint proteins [2,3], which have been shown to provide durable clinical responses in subsets of patients. In contrast, clinical outcomes from late-phase cancer vaccine trials have so far been disappointing. However, the combination of cancer vaccines with other therapeutic approaches [4] could enhance their efficacy and in this context the discovery of new vaccine targets for CRC is relevant.

Cancer/testis (CT) proteins are a category of proteins that hold particular promise as vaccine targets because of their restricted expression [2]. These proteins are expressed only in the testis or placenta which are immune privileged locations not accessible to the immune system, but they are overexpressed as a result of tumor development and may elicit specific CD8+ T-cell response because the adult immune system has not had prior exposure to these antigens.

IGF2 mRNA binding protein 3 (IGF2BP3) is an mRNA binding protein and cancer testis protein that regulates the translation of IGF2. IGF2BP3 and the other IGF2 mRNA binding proteins (IMPs) regulate IGF2 translation, stability and localization by binding to mRNA [5,6]. IGF2BP3 is a 580 amino acid protein with two RNA recognition motifs and four K homology domains, encoded by a 4350-bp mRNA transcript produced by the IGF2BP3 gene on chromosome 7p11.5 [7]. Overexpression of IGF2BP3 has been noted in numerous cancers including esophageal, lung, prostatic, gastric, pancreatic, hepatocellular and colorectal malignancies as well as sarcoma and melanoma [5,7,8–12]. In some cases, overexpression has been associated with a worse prognosis or more advanced disease [13–15].

Although prior investigators have assessed a group of colorectal adenocarcinomas for the presence of IGF2BP3 protein via IHC [15–17], a quantitative analysis of these tumors for IGF2BP3 mRNA expression in a sizable population of patients has not yet been carried out. It was the main purpose of the present study to do such an analysis and to compare tumor expression levels to that of adjacent normal colorectal tissue and testis tissue. In addition, tumor samples would also be assessed for the presence of IGF2BP3 protein via IHC.

## RESULTS

### Demographics, clinical data

A total of 84 pairs of normal and cancerous colorectal tissue were assessed in this study. There were 39 males and 45 females; average age was 67.5 +/− 14.4 years. The tumor locations were as follows: right, 42 (51%); sigmoid/rectosigmoid, 24 (29%); rectal, 11(13%); and transverse or descending colon, 7(6%). The study was limited to pathologic stage 2 and 3 tumors; there were 48 stage 2 (57%) and 36 stage 3 (43%) tumors.

### IGF2BP3 expression

**Figure 1:**
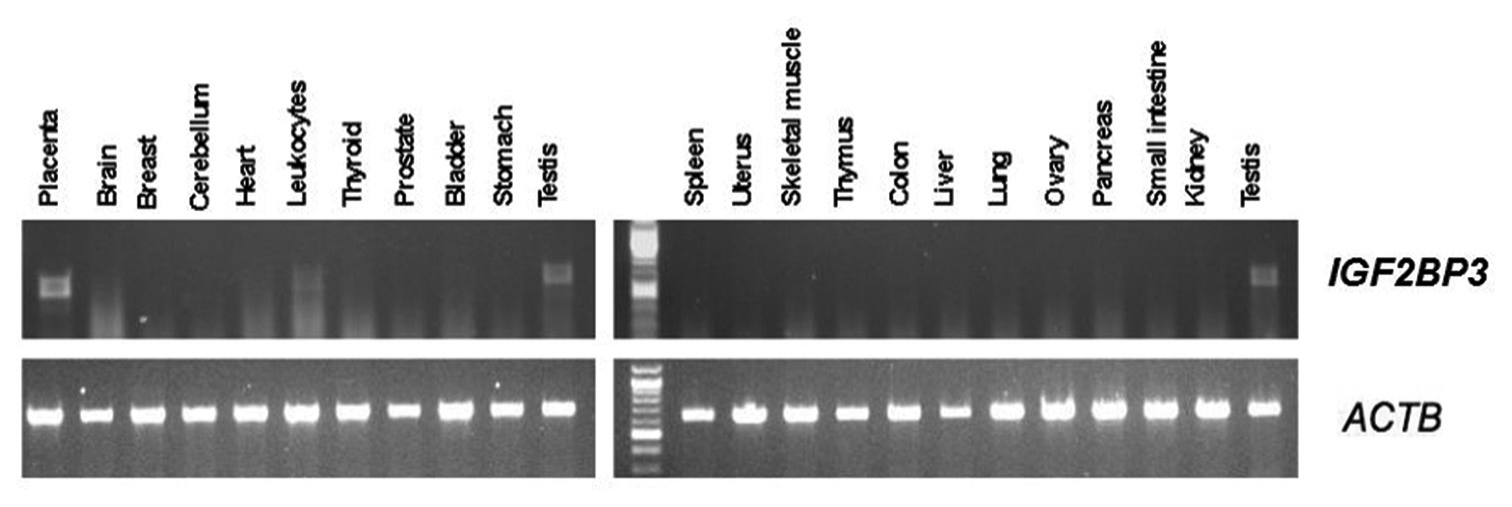
Semi-quantitative analysis of IGF2BP3 expression in pooled RNA from normal human tissues

To determine background values for normal tissues, we evaluated the expression of IGF2BP3 in 22 normal adult tissues using semi-quantitative RT-PCR (Figure 1). IGF2BP3 was expressed in placenta and testis. None of the other tissues assessed had significant IGF2BP3 mRNA expression with the exception of leukocytes, which demonstrated weak expression.

**Figure 2:**
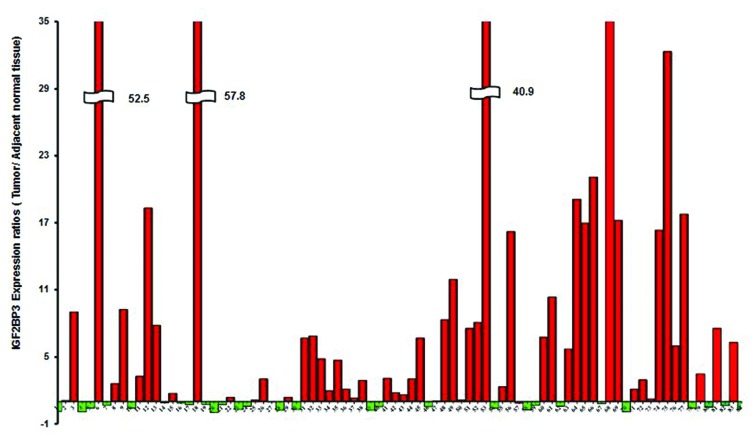
IGF2BP3 expression rations (Tumor/Adjacent tissue)

**Figure 3:**
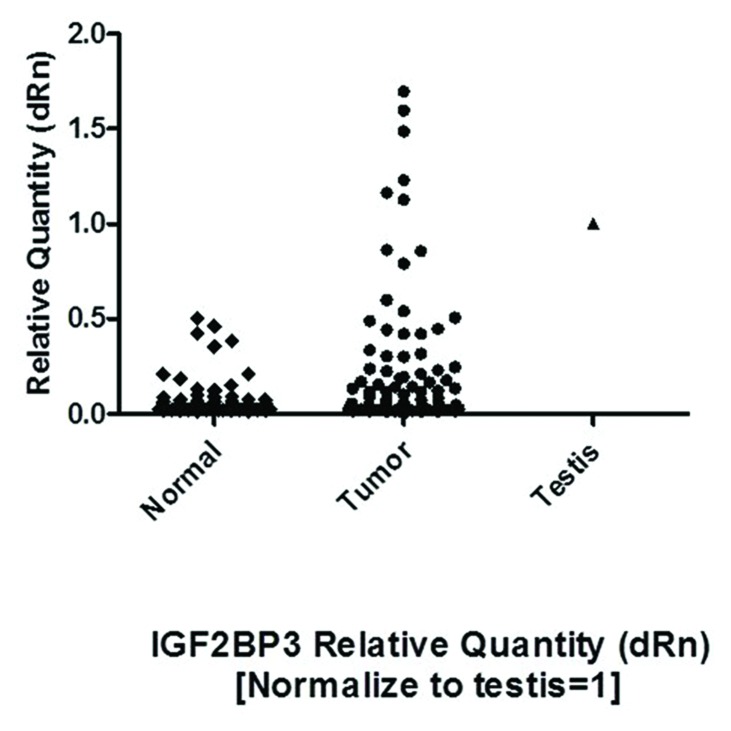
IGF2BP3 expression (dRn) in Colon tumor tissue, normal tissue (adjacent to tumor) and in Placenta (positive control)

All paired samples underwent quantitative PCR as described in the methods section. Of the 84 tumor specimens assessed via RT-PCR, 43 percent of the tumors met the criteria used for scoring positivity of cancer testis proteins, which comprises having expression levels that were greater than observed in normal colonic mucosa and also greater than 0.1 % of the testes expression level (expression levels over 100 in this study) [18]. When the expression results were analyzed on the basis of mucin producing vs. non-mucin producing tumors, 50 % of the former (3/6) and 43 % of the latter (34/78) had expression levels over 100. Using the less stringent criteria of tumor expression greater than normal mucosa [and at least equal to 0.1 % of the testes expression], 63% of the tumors were positive in this series (Figures 2, Figure 3).

**Figure 4:**
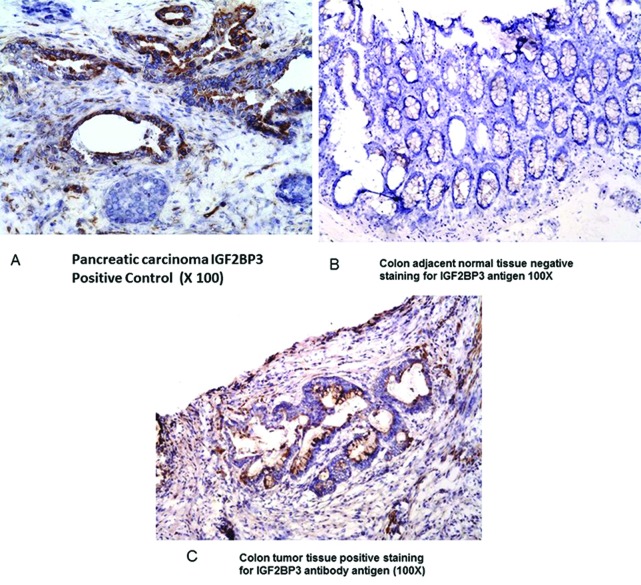
A Immunohistochemistry (IHC) staining of Pancreatic carcinoma (positive control), 4B: Normal colonic mucosa (negative control); 4C: colonic adenocarcinoma Purified rabbit anti human IGF2BP3 polyclonal antibody were used in IHC.

IHC was carried out on 46 paired tumor and normal tissues specimens that collectively covered the full range of RT-PCR protein expression. Positive IGF2BP3 cytoplasmic staining (1+ to 3+ intensity in >10% cells) was noted in 50% of the tumor specimens and 5% of the normal tissue specimens (1+ to 2+) intensity (Figure 4 A, 4B and 4C).

### Correlation between pathology results and expression levels

No significant differences were found in IGF2BP3 mRNA expression levels based on the stage of tumor or presence of lymph node metastases (n=36). There was a non-significant increase in mean expression levels in stage 3 tumors (0.125, CI 0.034–0.210) versus stage 2 tumors (0.073, CI 0.027–0.237) as well as in node-positive group (0.125, CI 0.034–0.210) versus node-negative group (0.073, CI 0.027–0.24). There was however, a significant increase in median IGF2BP3 expression level in women compared to men (p=0.042).

## METHODS

### Study population

The colorectal cancer specimens utilized for this study were taken from IRB approved tissue and data banks organized, managed, and maintained by the Sections of Colon and Rectal Surgery at New York Presbyterian Hospital, Columbia University Health Science Campus and Mount Sinai Roosevelt Hospital in New York City. Samples of tumor tissue and normal adjacent large bowel mucosa were taken from the resected specimens immediately following resection and were rapidly frozen.

Demographic data as well as the location of the cancer, the pathology results and the final tumor stage were obtained from the prospective data base. Tumor and paired normal tissue samples from 84 CRC patients (39 males,45 females); were studied. Among the study group 6 paired tissue samples were from patients with mucinous tumors.

### Tissue harvest

Tumor and normal mucosal samples were obtained from operative specimens after gross evaluation by a pathologist. The tissue samples were placed in separate standard Cryomolds (Tissue Tek, Sakura Finetek, Torrance, CA) which were filled with OCT (optimal cutting temperature) compound (Tissue Tek, Sakura Finetek, Torrance, CA) and placed in liquid nitrogen. The frozen tissue samples were stored in a −80°C freezer.

### Initial tissue evaluation

Prior to being included in this study, sections from candidate frozen tumor and normal colonic tissue blocks were taken at −20°C and assessed histologically to confirm the presence of tumor and the quality of each sample. This analysis was done at the Irving Cancer Center of Columbia University in New York City. Poor quality specimens and those with a paucity of tumor cells were not selected for the study.

### RNA extraction

Total RNA was isolated using Qiazol (Qiagen, CA), and purified using the miRNeasy Kit (Qiagen, CA). Briefly, sections of the frozen tissue block (previously embedded in OCT compound) were cut and placed into a 2ml Eppendorf tube, and then homogenized by TissueLyser II (Qiagen, CA) with 1ml Qiazol added. After the aqueous phase was recovered from the chloroform-derived phase separation, another step of acid phenol-chloroform extraction was performed with a phase lock gel tube (Qiagen, CA). RNA precipitation, column binding, on-column DNase digestion, washing and elution were performed according to kit instructions. RNA integrity was confirmed by agarose gel electrophoresis and the concentration was quantified by measuring OD260nm in BioPhotometer (Eppendorf, NY).

### Reverse transcription

The cDNA first strand reverse transcription was carried out with the ABI High Capacity RNA-to-cDNA Kit (Applied Biosystems, CA). Briefly, 1ug of total RNA and 10ul of 2X reverse transcription buffer and 1ul of 20X enzyme mix were incubated together in a total volume of 20ul at 37°C for 60 minutes followed by 95°C for 5 minutes and held at 4°C. The synthesized cDNA was stored at −20°C until further use.

### Quantitative PCR

Comparative quantitative PCR was performed with the Mx3005p real-time PCR machine (Stratagene, CA) using the QuantiTect SYBR Green PCR kit (Qiagen, CA). Briefly; PCR was carried out in 20μl total volume containing a final concentration of 1x reaction buffer, 300nM forward and reverse primers (Table 1) and 10ng cDNA (corresponding to the amount of input RNA). The PCR reaction steps were as follows; hot-start at 95°C for 15 min, and then 45 cycles of 95°C for 20 seconds, 55°C for 30 seconds, 72°C for 30 seconds, followed by dissociation curve measurement from 55 to 95°C. No template control (NTC) and no reverse transcription control (NRT) were included in every assay and all samples were done at least in duplicate. PCR data analysis was carried out with software Mxpro (Stratagene, CA). Comparative quantitative analysis was performed based on delta-delta Ct method using housekeeping gene GAPDH as the internal control. Results are expressed as relative quantity (dRn). Each plate contained amplification on testis and placenta cDNA templates which served as intra- and cross-plate calibrators.

**Table 1:** Real-time PCR Primer Information

Primer ID	Primer name	Sequence	Start	Tm	Location	Product size	SNP
386	IGF2BP3-AF	agttgttgtccctcgtgacc	1826	60.01	Exon14	145 bp	None
387	IGF2BP3-AR	agccttctgttgttggtgct	1970	59.91	Exon15		None

### Semi-quantitative RT-PCR

#### RNA samples

Pooled RNAs from normal tissues (testis, placenta, bladder, brain, breast, colon, small intestine, heart, kidney, leukocytes, liver, lung, skeletal muscle, ovary, pancreas, prostate, spleen, stomach, thymus, thyroid, uterus) were purchased from Clontech laboratories, Inc. (Palo Alto, CA) and Ambion, Inc. (Austin, Texas).

#### Reverse-transcription PCR

RNA samples (1 μg of commercial RNA from normal tissues) were reversed transcribed in a total volume of 20μl using Omniscript RT kit (Qiagen, Valencia, CA) according to the manufacturer 's protocol using oligo(dT)12-18 primer and RNaseOUT (Invitrogen, Carlsbad, CA). The cDNA was diluted five times with nuclease free water, and 3 μl of diluted cDNA were used in 25μl PCR reactions. For amplification, JumpStart^TM^REDTaq RedyMix (Sigma Aldrich, St. Louis, MO) and 10 pmol of each primer were used. Primers used for amplification were compiled from the literature or were designed using Primer3 software (http://frodo.wi.mit.edu/cgi-bin/primer3/primer3_www.cgi) [Primer forward: TCCCAGCTCTATCAGTCGGT; Primer reverse: TGTCTTTGGTTTGGC ATCTG; Amplicon size: 554 bp] Primers were designed to have an annealing temperature around 60°C and to encompass introns, thus allowing product discrimination in the case of genomic DNA amplification. Primer specificity was confirmed by aligning with the NCBI sequence database using BLAST http://blast.ncbi.nlm.nih.gov/Blast.cgi. The amplification protocol used was as follows: precycling hold at 95oC for three minutes followed by 35 cycles of denaturation at 95oC for 15 seconds, annealing for 30 seconds (10 cycles at 60oC, ten cycles at 58oC and 15 cycles at 56oC) and extension at 72oC for 30 seconds followed by a final extension step at 72oC for 7 minutes. PCR products were separated on 1.5% agarose gels stained with ethidium bromide.

### Tumor tissue selection for IHC

With the quantitative PCR results in hand, we selected 46 of our specimens representing a range of RT-PCR protein values from low to high for IHC in order to assess for the presence of IGF2BP3 protein in the tumor specimens and, thus, confirm the RT-PCR expression level results. The sections were cut at 5 mm-thickness and stained with hematoxylin and eosin (H&E). The slides were then reviewed by two pathologists to confirm the presence of tumor in each section. H&E stained slides were screened for optimal tissue and noncancerous tissue adjacent to tumor tissue. Normal colon tissue sections were also assessed after H & E staining for the presence of normal colonic tissue.

### IHC analysis

Immunohistochemistry was performed on fresh frozen slides (tumor/normal pairs) using standard IHC protocol. A fresh frozen sample of pancreatic adenocarcinoma was selected as the positive control. IHC staining was performed on 5 mm-thick sections of fresh frozen samples with purified rabbit anti human IGF2BP3 polyclonal antibody (Protein Tech Group, INC). The slides were incubated in H2O2 solution for 10 minutes at room temperature to block the endogenous peroxidase activity. Antigen retrieval was performed by steamer heating in 10mmol/L citrate buffer (pH 6.0). After epitope recovery, slides were incubated with primary antibody IGF2BP3 at 1:100 dilutions overnight at 4°C temperatures. Slides were washed and incubated with secondary anti-rabbit IgG (Vector laboratories, CA) at 1:200 dilution and tertiary streptavidin-peroxidase conjugate (ABC complex, Vector laboratories, CA). Slides were treated with chromogen “diaminobenzedine” for antigen detection. Counterstaining was performed with hematoxylin, dehydrated, cleared in xylene, and mounted on coated slides.

### Evaluation of immunohistochemical staining

Immunoreactivity was evaluated independently by two pathologists who assessed the immuno-staining. The percentage of neoplastic cells staining was determined (1 to 100%). IHC staining was considered “positive” when at least 10% or more of neoplastic cells stained positive on a scale of 1 to 3 (1+ weak, 2+ moderate and 3+ strong). IHC staining of 1+ intensity in less than 10% of neoplastic cells was considered negative, and IHC staining of 2–3+ staining in less than10% of cells was considered indeterminate.

### Statistical analysis

The demographic and clinical data are expressed as the mean and standard deviation of continuous variables whereas frequencies and percentages were determined for categorical variables. The Fisher exact test (for expected frequency of less than 5) and the Chi-Square test and were utilized for comparison of categorical variables such as the pathologic analysis. The t-test was utilized for comparison of continuous variables. A p value of less than 0.05 was considered statistically significant.

## DISCUSSION

The present study is the first to utilize RT-PCR to quantitatively assess IGF2BP3 mRNA expression in a reasonable sized population of colorectal cancers. One earlier study did RT-PCR analyzed a subset of 8 patients out of 203 tumors and demonstrated greater mRNA expression in tumors compared to normal tissues [16]. In the present study, as mentioned, 43% of the 84 tumors assessed met our criteria for positivity of Cancer Testis candidate proteins being assessed as potential vaccine targets (expression greater than the level noted in normal colon tissue and greater than 0.1% of the expression levels noted in testes tissue) [18]. The frequency of IGF2BP3 mRNA expression (50%) found in this study is actually one of the highest expression rates of a CT protein reported in colon so far. In our prior RT-PCR study, colorectal tumors were assessed for the expression of MAGE-A3 and CTAG2 by RT-PCR and the expression rates were found to be 28% and 17%, respectively [18]. Therefore, on the basis of tumor expression rate, IGFBP-3 holds some promise as vaccine target because it suggests that around 43% of CRC patients could benefit from an IBF2BP3-based vaccine. Of note, there is a clear precedent for using cancer-testis proteins as vaccine targets; clinical trials have been done using the cancer-testes proteins NY-ESO-1 and MAGE A [19–22].

To further assess a prospective cancer testis protein or any other protein as a vaccine target it is useful to assess preoperative plasma or serum specimens for the presence of antibodies to that protein to evaluate the occurrence of spontaneous immune response [23]. The finding of auto antibodies in a subset of patients would suggest that this protein can elicit an immune response that may help fighting the tumor. Therefore, the demonstration of serum auto-antibodies would raise the prospects of IGF2BP3 as a vaccine target. Although one shortcoming of the present study is that we were not able to determine auto-antibodies in the study population, highly immunogenic peptides derived from IGF2BP3 that can induce tumor-reactive and human leukocyte antigen (HLA)-A2 (A*02:01)-restricted cytotoxic T lymphocytes (CTL) were previously identified in lung cancer patients, suggesting that this protein can represent an important target for therapeutic vaccines [24].

Of note, in the present study, a non-significant increase in the mean IGF2BP3 mRNA expression levels was noted in the Stage 3 patients when compared to the Stage 2 patients. The same is true regarding the presence of lymph node metastases; a non-significant increase in the mean expression level was noted in the node positive group. Importantly, prior investigators who utilized IHC methods on larger populations (n=165–671) of patients have noted that IGF2BP3 expression was associated with a worse prognosis (advanced stage, lower DFS and overall survival) [15–17]. The relatively small size of the present study may explain why IGF2BP3 expression was not associated with more advanced disease. A larger RT-PCR study may clarify this issueand a future study that would also assess a broader range of colorectal cancer patients as regards tumor stage; Stage 1 and Stage 4 in addition to Stage 2 and 3 patients should be performed.

When a subset of tissue sections of the tumors in the present study were assessed via IHC, IGF2BP3 protein was noted in 50 percent of the cases. This result is in the same range as the RT-PCR mRNA result of 43% which is reassuring. Also, of note, the 43% RT-PCR result is closer to the IHC result than the 63% result obtained when the less stringent RT-PCR criteria is applied (expression greater than that noted in the normal colonic tissue) which support the use of the stricter criteria. Also, our IHC results are in the same general range as the results of several investigators who utilized IHC methods to assess for IGF2BP3. Li, et al in an IHC study of 203 colon cancers reported that 65% of tumors assessed expressed IGF2BP3 whereas Yuan et al, also using IHC methods, found an expression rate of 56% in a population of 165 colorectal tumors [16, 17]. In contrast, Lochhead et al, who analyzed 671 colorectal adenocarcinomas by IHC alone, reported that only 35% of tumor specimens expressed IGF2BP3 [15]. Presently, is not possible to reconcile Lochead et al's results with those of the other studies mentioned.

Some shortcomings of this current study include the fact that only stage 2 and 3 patients were considered. Further, as mentioned, no data is provided regarding serum titers of anti-IGF2BP3 antibodies for the study population. Also, this report supplies no oncologic outcome data (recurrence and survival rates). Thus, there is a need for larger RT-PCR studies that include patients with Stage 1 and 4 disease as well as antibody data and long term outcome data to shed further light on this topic.

## SUMMARY

In conclusion, this study is the first to perform RT-PCR for IGF2BP3 on a sizable population of colorectal cancer patients. Forty three percent of the tumors examined were found to express IGF2BP3 mRNA when compared to RT-PCR results obtained with each patients normal colonic tissue. Half of the subset of tumors that were assessed via IHC were found to express the IGF2BP3 protein. No significant difference in mRNA expression levels were noted for the Stage 3 vs the Stage 2 patients or node positive vs node negative tumors. Tumor expression of IGF2BP3 may hold promise as a tumor marker and/or vaccine target; however, larger studies are needed as well as determination of auto-antibody titers to IGF2BP3.

## References

[1] Kemeny N, Fata F (1999). Arterial, portal, or systemic chemotherapy for patients with hepatic metastasis of colorectal carcinoma. J Hepatobiliary Pancreat Surg.

[2] Lim SH, Zhang Y, Zhang J (2012). Cancer-testis antigens: the current status on antigen regulation and potential clinical use. Am J Blood Res.

[3] Sharma P1, Allison JP2 (2015). The future of immune checkpoint therapy. Science.

[4] Zamarin D, Postow MA2 (2015). Immune checkpoint modulation: Rational design of combination strategies. Pharmacol Ther.

[5] Nielsen J, Christiansen J, Lykke-Andersen J, Johnsen AH, Wewer UM, Nielsen FC (1999). A family of insulin-like growth factor II mRNA-binding proteins represses translation in late development. Mol Cell Biol.

[6] Liao B, Hu Y, Herrick DJ, Brewer G (2005). The RNA-binding protein IMP-3 is a translational activator of insulin-like growth factor II leader-3 mRNA during proliferation of human K562 leukemia cells. J Biol Chem.

[7] Müeller-Pillasch F1, Lacher U, Wallrapp C, Micha A, Zimmerhackl F, Hameister H, Varga G, Friess H, Büchler M, Beger HG, Vila MR, Adler G, Gress TM (1997). Cloning of a gene highly overexpressed in cancer coding for a novel KH-domain containing protein. Oncogene.

[8] Lu D, Vohra P, Chu PG (2009). An oncofetal protein IMP3: a new molecular marker for the detection of esophageal adenocarcinoma and high-grade dysplasia. Am J Surg Pathol.

[9] Li C, Rock KL, Woda BA (2007). IMP3 is a novel biomarker for adenocarcinoma in situ of the uterine cervix: an immunohistochemical study in comparison with p16(INK4a) expression. Mod Pathol.

[10] Wang T, Fan L, Watanabe Y (2003). L523S, an RNA-binding protein as a potential therapeutic target for lung cancer. Br J Cancer.

[11] Yantiss RK, Woda BA, Fanger GR, Kalos M, Whalen GF, Tada H, Andersen DK, Rock KL (2005). a novel molecular marker that distinguishes between benign and malignant lesions of the pancreas. Am J Surg Pathol.

[12] Zheng W, Yi X, Fadare O, Liang SX, Martel M, Schwartz PE, Jiang Z (2008). The oncofetal protein IMP3 A novel biomarker for endometrial serous carcinoma. Am J Surg Pathol.

[13] Barton VN, Donson AM, Birks DK, Kleinschmidt-DeMasters BK, Handler MH, Foreman NK, Rush SZ (2013). Insulin-like growth factor 2 mRNA binding protein 3 expression is an independent prognostic factor in pediatric pilocytic and pilomyxoid astrocytoma. J Neuropathol Exp Neurol.

[14] Yoshino K, Motoyama S, Koyota S, Shibuya K, Sato Y, Sasaki T, Wakita A, Saito H, Minamiya Y, Sugiyama T, Ogawa J (2014). Identification of insulin-like growth factor 2mRNA-binding protein 3 as a radioresistance factor in squamous esophageal cancer cells. Dis Esophagus.

[15] Lochhead P, Imamura Y, Morikawa T, Kuchiba A, Yamauchi M, Liao X, Qian ZR, Nishihara R, Wu K, Meyerhardt JA, Fuchs CS, Ogino S (2012). Insulin-like growth factor 2 messenger RNA binding protein 3 (IGF2BP3) is a marker of unfavourable prognosis in colorectal cancer. Eur J Cancer.

[16] Li D, Yan D, Tang H, Zhou C, Fan J, Li S, Wang X, Xia J, Huang F, Qiu G, Peng Z (2009). IMP3 is a novel prognostic marker that correlates *with* colon cancer progression and pathogenesis. Ann Surg Onc.

[17] Yuan RH, Wang CC, Chou CC, Chang KJ, Lee PH, Jeng YM (2009). Diffuse expression of RNA-binding protein IMP3 predicts high-stage lymph node metastasis and poor prognosis in colorectal adenocarcinoma. Ann Surg Oncol.

[18] Shantha Kumara HMC, Grieco MJ, Caballero OL, Su T, Ahmed A, Ritter E, Gnjatic S, Cekic V, Old LJ, Simpson AJ, Cordon-Cardo C, Whelan RL (2012). MAGE-A3 is highly expressed in a subset of colorectal cancer patients. Cancer Immun.

[19] Davis ID, Chen W, Jackson H, Parente P, Shackleton M, Hopkins W, Chen Q, Dimopoulos N, Luke T, Murphy R, Scott AM, Maraskovsky E, McArthur G, MacGregor D, Sturrock S, Tai TY, Green S, Cuthbertson A, Maher D, Miloradovic L, Mitchell SV, Ritter G, Jungbluth AA, Chen YT, Gnjatic S, Hoffman EW, Old LJ, Cebon JS (2004). RECOMBINANT NY-ESO-1 protein with ISCOMATRIX adjuvant induces broad integrated antibody and CD4+ and CD8+ T cell responses in humans. Proc. Natl. Acad. Sci. USA.

[20] Chen Q, Jackson H, Parente P, Luke T, Rizkalla M, Tai TY, Zhu HC, Mifsud NA, Dimopoulos N, Masterman KA, Hopkins W, Goldie H, Maraskovsky E, Green S, Miloradovic L, McCluskey J, Old LJ, Davis ID, Cebon J, Chen W (2004). Immunodominant CD4+ responses identified in a patient vaccinated with full-length NY-ESO-1 formulated with ISCOMATRIX adjuvant. Proc Natl Acad. Sci. USA.

[21] Marchand M, van Baren N, Weynants P, Brichard V, Dréno B, Tessier MH, Rankin E, Parmiani G, Arienti F, Humblet Y, Bourlond A, Vanwijck R, Liénard D, Beauduin M, Dietrich PY, Russo V, Kerger J, Masucci G, Jäger E, De Greve J, Atzpodien J, Brasseur F, Coulie PG, van der Bruggen P, Boon T (1998). Tumor regressions observed in patients with metastatic melanoma treated with antigenic peptide encoded by MAGE-3 and presented by HLA-A1. Intl J Cancer.

[22] Jäger E, Gnjatic S, Nagata Y, Stockert E, Jäger D, Karbach J, Neumann A, Rieckenberg J, Chen YT, Ritter G, Hoffman E, Arand M, Old LJ, Knuth A (2000). Induction of primary NY-ESO-1 immunity: CD8+ T lymphocyte and antibody responses in peptide-vaccinated patients with NY-ESO-1+ cancers. Proc. Natl. Acad. Sci. USA.

[23] Beeton-Kempen N1, Duarte J, Shoko A, Serufuri JM, John T, Cebon J, Blackburn J (2014). Development of a novel, quantitative protein microarray platform for the multiplexed serological analysis of autoantibodies to cancer-testis antigens. Int J Cancer.

[24] Tomita Y, Harao M, Senju S, Imai K, Hirata S, Irie A, Inoue M, Hayashida Y, Yoshimoto K, Shiraishi K, Mori T, Nomori H, Kohrogi H, Nishimura Y (2011). Peptides derived from human insulin-like growth factor-II mRNA binding protein 3 can induce human leukocyte antigen-A2-restricted cytotoxic T lymphocytes reactive to cancer cells. Cancer Sci.

